# Patent information retrieval: approaching a method and analysing nanotechnology patent collaborations

**DOI:** 10.1007/s11192-017-2325-y

**Published:** 2017-03-06

**Authors:** Sercan Ozcan, Nazrul Islam

**Affiliations:** 1 Portsmouth Business School, University of Portsmouth, Portsmouth, UK; 2 The University of Exeter Business School, University of Exeter, Exeter, UK

**Keywords:** Tech-mining, Patent information, Search query, Collaborations, Empirical analysis, Nanotechnology

## Abstract

Many challenges still remain in the processing of explicit technological knowledge documents such as patents. Given the limitations and drawbacks of the existing approaches, this research sets out to develop an improved method for searching patent databases and extracting patent information to increase the efficiency and reliability of nanotechnology patent information retrieval process and to empirically analyse patent collaboration. A tech-mining method was applied and the subsequent analysis was performed using Thomson data analyser software. The findings show that nations such as Korea and Japan are highly collaborative in sharing technological knowledge across academic and corporate organisations within their national boundaries, and China presents, in some cases, a great illustration of effective patent collaboration and co-inventorship. This study also analyses key patent strengths by country, organisation and technology.

## Introduction

Patenting activities and knowledge diffusion in high-tech sectors are being increasingly driven by collaborative, international and technology-based new entrants, such as spinoffs and SMEs (Gredel et al. 2011; Qian and Chen 2011). Generally, diffusion of technologies is highly dependent on a market structure and, currently, the demand for new inventions drives the generating of the increasing number of patents. However, in emerging technologies, such as nanotechnology, this may not be the case as the demand may need to be created or the process needs to be supported by actors (e.g. government; academics in research institutes; corporations) so that technology diffusion is efficient. Many researchers believe that the knowledge of technology development and diffusion can be gained through patent analysis, as patent documents provide a valuable resource of information to analyse a technological field or an innovation system if the data are analysed systematically (Choi and Park 2009; Lee et al. 2011). Some of the reasons why patent analyses are pursued include the discovery of promising technologies; the assessment of technological advances and new trends, or helping organisations in their strategic decision-making (Firat et al. 2008).

Nanotechnology is a growing area and is considered to be an emerging technology (Linton and Walsh 2008; Islam and Miyazaki 2009). In considering nanotechnology patent information; mining and its management, one of the key issues is to use an expedient patent database in terms of the required size and the coverage of patents. The problem with collecting the required nanotechnology-related patents is that there are many patents that include the nano-related unnecessary and unrelated terms within the nanotechnology patent category. As a result, there is a possibility of obtaining these unrelated patents with the nano-patented inventions. This research highlights the challenges incurred with collecting the accurate patents in the nanotechnology field and proposes an improved method on how the accurate patents are collected. For this purpose, various patent databases were compared to find the best offering in terms of, among others, the number of patents offered, and the coverage of patent authorities. As such, the validity and reliability of the patent collection method is examined and the strengths and weaknesses of each patent database are also considered. For nanotechnology information retrieval, some criteria were crucial; namely, the patent authority coverage; the maximum hit list; the availability of various patent database export options and the maximum allowed export quantity of patent documents.

A review of the previous literature indicated that there are certain limitations to the existing research. These limitations can be divided into those concerned with the methodology applied and the type of research. For example, Huang et al. (2011) categorised lexical and patent classification queries by analysing related methodological studies. Porter et al. (2008) and Mogoutov and Kahane (2007) have used lexical queries to gather all patents with ‘nano’ terms, which resulted in around 140,000 patents that revealed many unrelated patents. Therefore, many challenges still remain in the processing of patent information, i.e. explicit technological knowledge documents, which demand an improved approach. Given the limitations and drawbacks of the existing approaches (Huang et al. 2003; Scheu et al. 2006; Porter et al. 2008), this research sets out to develop an improved method which uses a combination of both patent classification codes and lexical queries. This approach helps accurate nanotechnology patent information retrieval. Details are presented in “Research methodology” section.

This study examines the structure and significance of patenting activity to understand the related determinants that affect the nano-technological knowledge diffusion process. Using nanotechnology patent information, the research investigates the nano-knowledge management focusing on patent collaborations and the patent strengths by technology, actors, and country. A comparative analysis is also presented so that governments, academics and corporations can benefit from the research findings.

## Background and theoretical framework

There are plenty of patent studies that focus on the association amongst technological advancement and economic progression (Greif 1992; Ma et al. 2009; Hidalgo et al. 2010); the research and innovation developments in a global context (Abraham and Moitra 2001; Faber and Hesen 2004; Encaoua et al. 2006; Wu and Lee 2007), and the stage of technology development in a particular sector (Bachmann 1998; Trappey et al. 2011; Tseng et al. 2011). In some studies, the relationship between key actors are analysed within a particular innovation system (Waguespack and Birnir 2005; Tödtling et al. 2009; Dangelico et al. 2010). Over the past few years, various researches have been attempted on nanotechnology information management (for example, three top-ranked journals called ‘*Research Policy’*, ‘*Technological Forecasting and Social Change’* and ‘*Technovation’* were published with their special issues on nanotechnology).

The most relevant studies conducted in recent years focusing nanotechnology patent analyses are: Shapira et al. (2011) focus on an overview of corporate entry into nanotechnology through patents and publications and nanotechnology innovation factors in the shift to commercialization. Chien et al. (2011) present the data envelopment analysis (DEA) approach to evaluate a nation’s technology efficiency and effectiveness in Asian countries. The highly cited, earlier work of Huang et al. (2003) completed a similar practice by presenting a longitudinal patent analysis on nanotechnology patents. Their work included content map analysis and citation network analysis by obtaining the required data from individual countries, institutions and technology fields. Nanotechnology can be classified as a science-based cluster (OECD 1997) which is highly R&D and patent-focused and is likely to have a close relationship with the public research sector (i.e. universities, government research bodies, etc.). This is due to their requirement for basic research and so it is essential for the public research sector to become involved for there to be an effective innovation structure. The system of innovation literature helps analysing patent collaborators and key technology strengths in nanotechnology. The innovation system comprises of the linkages and flow of information among actors, such as inventors and organisations in terms of innovative processes (Lundvall 1992; Liu and White 2001; Doloreux 2002; Yim and Kang 2008; Guan and Chen 2012a, b). Feldman et al. (2006) examined innovation systems and the involvement of academia in the commercialisation process to identify technology transfer in biomedical research. The authors compared different innovation systems and the influence of universities in this particular field, focusing on issues, such as public funding and the commercialisation of science.

After comparing different models, their work indicated that actors are one of the key determinants when considering the differences between national innovation systems. One of the most important influences of national innovation system (NSI) in the innovation management field is its attempt to categorise actor and institutions’ functions. There are different categorisations of actors within NSI studies and (Nelson 2009) categorises them into three groups: R&D systems, governments and universities. According to OECD (1997), NSI actors are primarily private enterprises, universities and public research institutes. Looking at both classifications, three actors can be identified as: (1) organisations that are involved in research; (2) organisations involved in industry and R&D; (3) governmental organisations. The triple helix model is another popular model that is used in similar studies. It scrutinises the relationships between actors within an innovation system. The triple helix model is one of the innovation models that present the manifold, mutual relationships at various stages of knowledge-capitalization processes (Etzkowitz and Leydesdorff 2000). The triple helix model denotes the university–industry–government relationship as one of relatively equal, yet interdependent, institutional spheres which overlap and in which institutions intermittently assume and exchange roles (Etzkowitz and Leydesdorff 2000). In comparison to other models, the triple helix would be the model that differentiates actors’ relationships based on their overlapping functions.

**Fig. 1:**
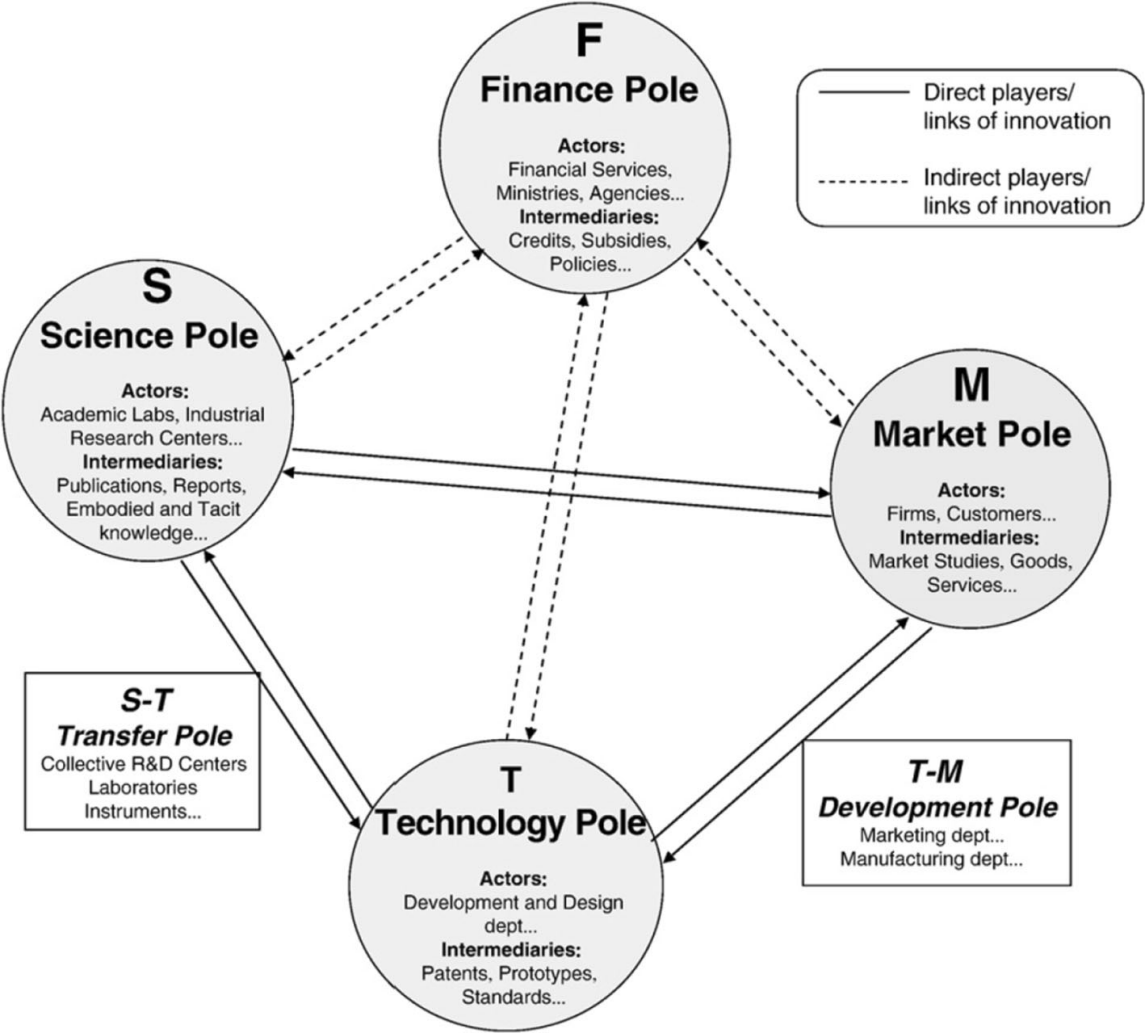
Techno-economic network (TEN) framework (adapted and modified from Callon and Bell 1991)

Consoli and Patrucco (2008) state that innovation requires the coordination of distributed knowledge amongst different organisations as a collective process. In their comparative study of the UK and Italy, they illustrate the significance of organisational responsiveness in stimulating collective innovation processes. Their findings show differences between the structures of both networks and demonstrate that these differences are related to how the actors are integrated. Another framework that illustrates the roles and linkages of actors is the techno-economic network (TEN; see Fig. 1), which is a useful framework to analyse the systems of innovation in a comprehensive manner for a chosen sector (Callon and Bell 1991). There are three major poles within the TEN, such as the technology pole; the science pole and the market pole. Another minor pole which appeared within this framework is the finance pole, due to its indirect players or links to innovation. Each of these poles is categorized by the type of actors and intermediaries in regards to its duties. As illustrated in Fig. 1, intermediaries vary in terms of tangible and intangible resources for those actors within TEN. Moreover, it presents how these poles are linked to each other in terms of their direct or indirect linkage and which intermediaries they are linked by, such as the transfer pole (between the science pole and the technology pole) and the development pole (between the technology pole and the market pole).

Nanotechnology is not a sector, and is considered a general purpose technology (GPT): it either enables technology or is disruptive to it. Nanotechnology is a highly dispersed, multi-disciplinary area that is distributed throughout a number of sciences and disciplines. It may be easy to identify nanotechnology field boundaries in terms of its national externality; however, it may be difficult to identify sectorial or technological boundaries. In light of previous studies as well as critiques of SI studies in relation to accepted boundaries, it may not be correct to apply NSI-, SSI- or TSI-based boundaries to a nanotechnology innovation system (Nano-SI). Even though the nanotechnology field has diffused into certain technologies (electronics) and materials (semiconductors), it is not possible to accept that a combination of certain TSIs may form the boundaries of Nano-SI, since nanotechnology-related technologies and activities can be a small or a large part of those TSIs. One assumption that can be made is that the boundary of a Nano-SI may be based on combinations of TSI technological domains.

Considering the nanotechnology innovation system, it would be expected that there would not be national boundaries and its whole system would be at an international level. There are two main reasons that lead us to these notions. Firstly, there are active, global players within the nanotechnology system that are known from previous studies (Meyer 2001; Cunningham 2011; Shapira et al. 2011) and it would be expected that these organisations would have linkages at the global level in terms of their research activities and their participations with other research institutes. Secondly, nanotechnology is an emerging field and some other studies (Islam and Miyazaki 2009) illustrated that this multidisciplinary field has an impact on various technologies. As a result, TEN framework provides a simple, comprehensive and flexible conceptual framework, as this study aims to use patent information in order to investigate the linkages of actors with the technology pole. Accordingly, it is possible to look at the linkages and collaborations between the S–T and T–M poles. By analysing all patent information using the proposed taxonomy, it would be possible to see how current technology sources are generated and how these actors are linked to each other in terms of shared patents.

## Patent information retrieval: structure and significance

The function of patents (generating secured technologies or leading to organisational collaborations) may not be true as it may hinder or support innovation processes depending on various conditions, such as the inimitable and exclusive patents that are the core technologies (Fontana et al. 2006; Motohashi and Muramatsu 2012). For this purpose, the structure of patenting activity should be examined thoroughly to understand the related determinants that affect this process. By exploring the changes in a particular patent data, it is possible to evaluate many aspects of technological change. Patent analysis is relatively significant in various contexts but there are some limitations to these studies. This is due to the fact that not all patented inventions are commercialised, and not every innovation has a fundamental influence on technological or economic value. There is a conflict between the generally accepted positive influences of patents on innovation and the contrasting notion that patents have a negative effect on technology diffusion, resulting in unfair competition (Andolfatto and MacDonald 1998; Saint-Paul 2004). Questions in relation to patent activities and the diffusion of technology can be raised, e.g. whether patented inventions support the diffusion of technology or the national barriers for other organisations to use that specific technology (IP, marketing strategies or lengthy organisational learning curves, etc.) so it deters or obstructs the diffusion process. The key question that this paper asks is related to the patent classifications for the nanotechnology field as they were introduced in 2004; are still in their development stage and so this field could benefit from bibliometric analyses to help to classify the sub-domains of nanotechnology.

In regard to the role of the key actors in innovation systems, there are important responsibilities for governments, research institutes, corporations and inventors as they are investigated in many researches (Chiang 1995; Sorenson et al. 2006). In a specific country or a technology, the key actors who play a central role in the technology diffusion process may be different. The linkage between key actors in one research domain may vary to that of another and these different linkages may lead to more or less productive innovation systems. Analysing patenting activities at a country level with a particular focus on a specific technology would be one way to observe which settings of the innovation process are more productive and so improve the effectiveness of patenting systems and the diffusion of new inventions. In the case of Japan, when stronger patent rights were granted to their owners, the result was a more effective technology transfer and licensing of inventions. However, it is not possible to assume that this would hold true for other countries. It is essential to analyse the patenting activities at a national level to see the effectiveness of various policies and regulations (OECD 2004).

The relationship between large organisations, SMEs and spinoffs are playing an increasingly significant role in the globalisation of innovation (Gredel et al. 2011; Qian and Chen 2011). Many changes in the collaborative structure between the various actors in an innovation system lead to the interconnection of higher numbers of and more diverse actors. Increased security of patent authorisation and the profitability of patenting encourage inventors to participate more in the patent generation system. The necessary high costs of R&D and the risks of unsuccessful commercialisation attempts are encouraging companies to participate in innovation systems (Forero-Pineda 2006; Lichtenthaler et al. 2009). Even multi-national companies are focusing on their key capabilities and obtaining complementary technologies from other organisations, such as universities, institutes and their collaborative firms (Maine et al. 2012). As a result, there has been a rapid rise in the number of companies that are collaborating in patenting activities and the linkages between collaborative organisations are getting stronger.

Academic actors play a key role within the patent generation process as, nowadays, they frequently collaborate with large companies and are also fully or partly supported by public funds (Grimaldi et al. 2011). The increase in academic patenting greatly supports the technology diffusion process because the core notion underpinning technology transfer from universities is the commercialisation of the research results (Goldfarb and Henrekson 2003; Crespi et al. 2011). Some governments are aiming to motivate academic organisations by transferring patent ownership from the government to academic organisations, which eases the technology transfer process by increasing legal certainty and reducing transaction costs (OECD 2004). Growing interrelationships amongst countries in the context of collaboration within different aspects of technology have fostered the usage and implementation of patents with the purpose of ensuring funds are invested in innovation and increasing the dissemination of technology (Senker 1996). In addition to that, increased competition in some markets has resulted in companies relying on granted patents and this has motivated them to focus on research activities. High R&D investments support the increase in the number of granted patents but cannot entirely throw light on the increase of innovations.

## Research methodology

**Fig. 2:**
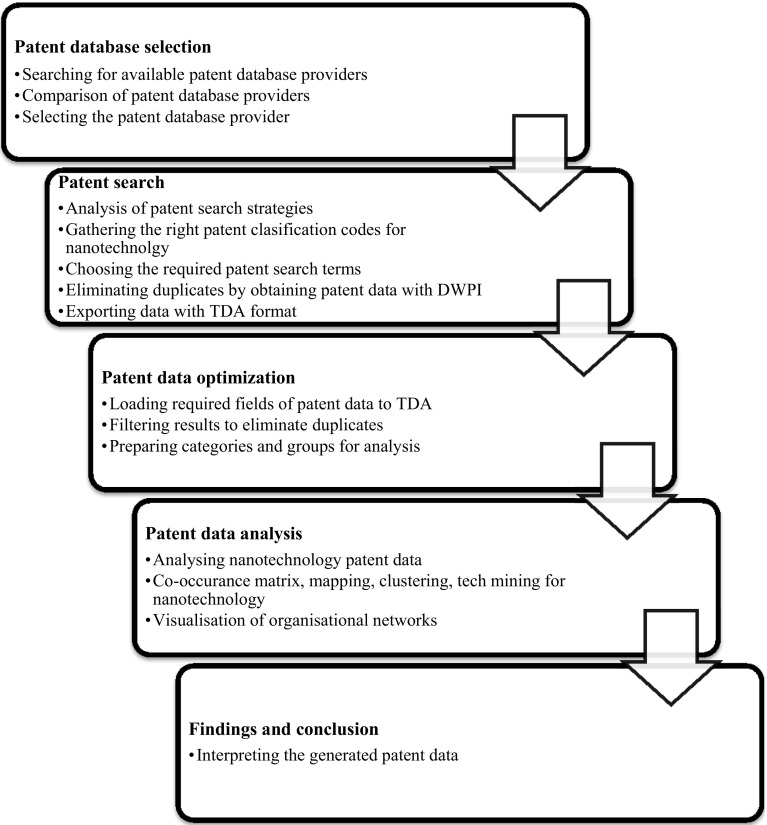
The outline of research process

In general, gathering the valid patent data; the efficient analysis of large data sets as referred as “big data” by many scholars nowadays and handling and interpreting the outcomes of the analysis are crucial for the accuracy of the results. In the research methodology, sampling and its link to generalizability and quality of implications is vital to the whole research process (Collins et al. 2007). One of the weakness of the current bibliometrics or scientometrics literature found to be performing analysis on the inaccurate data set which leading to inaccurate results. Many scholars are found to be using some generic search queries where they end up retrieving wrong data and hence wrong results. Since nanotechnology is a highly dynamic, emerging field; the progress of patents, innovations and industry is rapidly changing, this causes even higher uncertainty. Being able to conduct a high quality study in such field would prove that similar approach can be used in any other field and so there is great replicability. For this purpose, this research develops an improved method for searching and extracting accurate nanotechnology patent data. For analysing the data, the tech-mining method was applied—proposed by Porter and Cunningham (2005)—which analyses relations between actors and technologies; identifies the key patent strengths within a given innovation system. The subsequent analysis was performed using dedicated tech-mining software, the Thomson data analyser (TDA); automating mining and clustering of terms occurring in article abstracts and article descriptors, such as authors, affiliations or keywords. The outline of methodology and the general process can be seen, as shown in Fig. 2. In general, gathering the valid patent data; the efficient analysis of large data sets, and handling and interpreting the outcomes of the analysis are all crucial for the accuracy of the results.

### A comparative illustration of patent databases

One of the key issues for a study as in this field is to use an expedient patent database in terms of the required size and the coverage of patents. For this purpose, various patent databases were compared to find the best offering in terms of the number of patents offered and the coverage of patent authorities. Strengths and weaknesses of each patent database are considered and illustrated in Table 1.

**Table 1:** A comparative illustration of patent databases

Patent database	Delphion	MicroPatent PatentWeb	PatBase	Thomson innovation
Database provider	Thomson reuters	Thomson reuters	Minesoft Ltd; RWS group	Thomson reuters
Tool type	Patent search systems, non-patent data provider, commercial/pay databases	Patent search systems, commercial/pay databases	Patent search systems, commercial/pay databases	Patent analytical tool, patent search systems, non-patent data provider, commercial/pay databases
Interface language	English, Japanese	English	English, Japanese	English, Japanese
Patent authority coverage	4 (US, EP, WO/PCT, DE)	6 (US, EP, WO/PCT, DE, FR, GB)	18 (US, EP, WO/PCT, JP, BE, BR, CH, CN, DE, DK, ES, FI, FR, GB, IN, KR, SE, TW)	8 (US, EP, WO/PCT, JP, DE, GB, FR, KR)
Bibliographic: patent authority coverage	INPADOC and DWPI	GB, FR, DE	INPADOC, TH, TT, UZ	INPADOC and DWPI
Machine pre-translated data	No	No	Yes, at least AR, BR, CN, DE, EP, ES, FR, IT, JP, KR, MX, TW	Yes, JP (machine-assisted translations), CN (hand translations), KR (machine translations)
Corporate tree data	Yes, corporate data is from 1790 analytics	No	No	Yes, corporate data is from 1790 analytics
Non-patent coverage	Yes	No	Yes	Yes
Special indexing	DWPI	No	No	DWPI, Inspec, and ISI web of science
Derwent WPI family coverage	Yes	No	No	Yes
Backward citations	Yes	Yes	Yes	Yes
Forward citations	Yes	Yes	Yes	Yes
Citation data coverage	US	US, WO/PCT, EP, GB, DE; partial coverage for FR, JP	US, EP, WO/PCT, JP, AP, AU, BE, BG, CH, CY, CZ, DE, DK, EA, ES, FI, FR, GB, GR, IT, KR, LU, NL, NO, SG, TR	US from 1971; WO/PCT from 1978; EP from 1978; EP from 1978; DE from 1988; GB from 1978; JP from 1994; KR from 2008
Original US class	Yes	No	No	Yes
Patent classification data	US class, IPC, ECLA, JP F-terms, any national class in the INPADOC	US Class, IPC, ECLA	US class, IPC, ECLA, JP F-terms, Dekla, Locarno	US Class, IPC, ECLA, JP F-terms, Locarno
Max hit list size	500	20,000	100,000	60,000
Family sorting	No	Yes (INPADOC)	Yes (INPADOC)	Yes (INPADOC or DWPI)
Formats for export data	CSV, Derwent analytics, ResearchSoft (RIS), tagged (TAG), XML (all in one file, or one file per patent)	BizInt smart charts^®^ (BPD), CSV, HTML for “family reports,” PDF, ResearchSoft^®^ (RIS), TSV	BizInt smart charts^®^ (BPD), DOC, CSV, HTML, patent iNSIGHT pro, PDF, RTF, VantagePoint, XLS, XML, INTELLIXIR	BizInt smart charts^®^ (BPD), CSV, excel 2007 (XLSX), HTML, PDF, TSV, TXT, ResearchSoft^®^ (RIS), RTF, XML, spotfire, Thomson data analyzer
Keyword analysis	Yes	No	Yes	Yes

The data is taken from intellogist.com

As shown in Table 1, some criteria were crucial, namely the patent authority coverage, maximum hit list, availability of various patent database export options and the maximum allowed export quantity of patent documents. This is due to the fact that the required patent database was large and exceeded some of the patent database providers’ maximum allowed patents document export option. Delphion and MicroPatent provide a limited number of patent authorities. While their competitor, PatBase, does have a significant number of patent authority coverage but there are service restrictions in terms of search hit list and the number of patent documents that would limit the potential data size. The most common patent data providers for such studies where large data is required with high coverage are Thomson innovation and Patbase. PatBase offers the highest number of patent authority coverage and the greatest hit list of 100,000. However, the export option is limited to 20,000 records per month and this would be a drawback if the required patent database is higher than 20,000, giving it the same drawback as MicroPatent. Thomson innovation has a significant number of patent authority coverage but it is smaller than Patbase’s coverage. As a result of this comparison between various patent database providers, Thomson innovation was the preferred patent database as the required large data set could be gathered and analysed by TDA software.

### A method for nanotechnology patent information retrieval

One of the biggest challenges in a patent analysis is to gather the required patent data by selecting the appropriate terms for the search so that the data set includes the relevant patents and excludes unnecessary patents, thus increasing the validity of the research. Moreover, it is an even greater challenge if the analysed field is an emerging technology and there are many similar terms that are used by other technologies. In the case of nanotechnology, the USPTO created a nanotechnology patent class labelled 977 in 2005 as a cross-reference collection, and its sub-categories, to gather all the nanotechnology related patents within this category. Class 977 presents additional collections for patent searches, but it is not very useful for categorizing patents as a basis for assigning applications because nanotechnology related US patents are only classified in class 977 as a secondary or a cross-reference classification; they are not primary classifications. For primary classifications, B82 by IPC is used and this classification is very helpful if nanotechnology patents are required to be analysed in terms of nanotechnology’s sub-domains or sectors. This was a useful approach considering the consistency of the nanotechnology related patent analysis, as this field is very dispersed among various fields such as electronic, biological and robotic applications. The negative aspect of this new nanotechnology patent classification is that nano-related inventions were patented first in the 1980’s, so many patent authorities, such as USPTO assigned teams, had to reclassify the records of patents granted previously to the established nanotechnology patent classification because at the time these classifications were introduced by patent authorities, many nanotechnology related patents had been introduced with different patent classifications. However, the majority of existing nanotechnology related patents have been reclassified into their respective patent classifications and new nanotechnology patents are classified into the required classification. The main problem in finding nanotechnology-related patents is that there are some patents within the nanotechnology class that are not related to the nanotechnology field (e.g. the following patents have been classified under the patent code B82; however, they are not really at the nano level. Please see the patent documents: WO2001097295 A3, EP1688735 B1 and WO2012047042 A3).

Various approaches are followed by patent analysts and researchers in this field. There are many limitations and drawbacks in terms of the search terms that are used and the nanotechnology patents which are obtained. There are two main approaches in this field. One of the approaches is to use all the required nanotechnology related terms such as nanotube, nanowire and nano-sensors in the patent search and to try to get the highest possible hit list as a result. This type of search may face two major problems. The first one is that the researcher may not cover all the required nano-terms, and, as a result may not be able to access all the required nanotechnology related patents, for example colloidal crystals, quantum dot and fullerene do not include the term ‘nano’, but they involve nanotechnology related patents. Another issue with this type of research is that there are many patents that mention nanotechnology related materials within patent documents that are not for a nanotechnology invention. For example, if the details of some of the patents are analysed, it can be seen that the nanotechnology related term is used in the description of a non-nanotechnology patent that states the invention can also be used with one type of nanomaterial such as nanotubes. As a result, it is possible to include unnecessary patents and exclude necessary patents in the analysed patent data set. The second common approach in nanotechnology related patent analysis is to obtain all the patents that include terms that start with prefixes, such as ‘nano’ or ‘quantum’, by using Boolean search operands such as nano* OR quantum* and excluding all the unnecessary patents from the result which include terms such as ‘nanosecond’ and ‘nanometre’. The problem with this approach is that there are many nanotechnology related patents that include those unnecessary terms, for instance there are many nanotechnology patents that include both ‘nanowire’ and ‘nanosecond’. This is due to fact that there are many nano related unnecessary terms and some unrelated patents, such as micro level patents that are included within the nanotechnology patent category. For example, large companies such as IBM have many electronics related patents that have nanotechnology related terms and ‘nanosecond’ in their patents, so those patents would be eliminated as well. As a result, there is a possibility of obtaining unrelated patents with the nano-patented inventions.

Huang et al. (2011) analysed patents and publication research approaches, and categorized them into two broad strategies: lexical queries and patent classification queries. Authors mention both the advantages and the disadvantages of these two forms of query. Porter et al. (2008) used lexical queries to gather all patents with ‘nano’ terms including those patents that have unrelated terms such as ‘nanosecond’. Our proposed method uses a combination of both the patent classifications code and lexical queries. The reason why both approaches are followed is because—as is mentioned in Scheu et al. (2006) study—only using patent codes has a weakness in that unrelated patents appear in the patent data due to their wrong classification. Also, using only lexical queries—as suggested by Porter et al. (2008)—resulted in almost 140,000 patents, among which were found many unrelated patents after reviewing the samples from the collected data. However, Porter et al. (2008) lexical search query appears to be most reliable for publication data retrieval. Afterwards, the DWPI (Derwent patent index) was used to exclude patents that appeared more than once in the search results.

For the nanotechnology case, the following search terms are used:

[AIOE = (B82*) OR FIC = (B82*) OR UCC = (977*)] AND ALLD = (nano* OR quantum* OR Qdot OR Qubit OR atom* OR probe OR epitax* OR fullerene* OR thin ADJ wire* OR thin ADJ film* OR buckyball* OR scanning ADJ microscope* OR tunnelling ADJ microscope* OR scanning ADJ electron* OR bionano* OR bio-nano* OR gCNT* OR Peapod* OR CSCNT* OR CNT* OR g-CNT* OR colloidal ADJ crystal*).

**Fig. 3:**
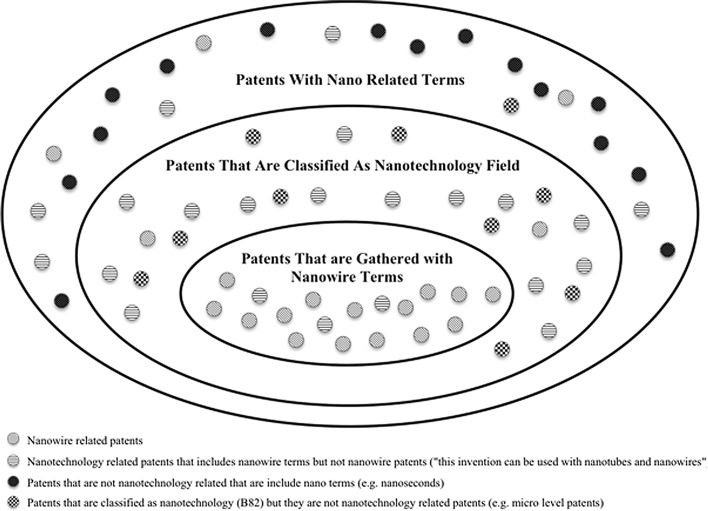
Illustration of collected patent data

The validity and reliability of this patent collection method is illustrated in Fig. 3. Figure 3 shows how the required patents are systematically collected. Four different nano-related patent categories are introduced. The first comprises of those nanotech-related patents that are required to be collected. The second types of patents that are mentioned in Fig. 3 are those nanotechnology-related patents that include nanotech-related terms but are not really nanotech-related patents. To give an example, there are many documents that mention nanotech-related terms, such as, “this new material also can be used with nanotubes, nanowires and nanotech,” but the patent is not really related to nanotech patents. This group is very difficult to eliminate from the patent data as it contains cases categorized under nanotechnology-related categories, so the only way of eliminating these patents is to examine the patents individually. The third group are those patents that include ‘nano’ terms but are not nanotechnology-related patents, such as ‘nanosecond’ or the ‘iPod nano’. Patents in this group are easy to eliminate using this patent collection method as they are using nanotechnology classifications; nanotech terms and this means that they are double-checked. The last patent type comprises of those patents that are classified under the nanotechnology category, such as B82 or 977, but are not nanotechnology-related patents. There are many micro-structural-related patents under these categories and the main problem with these is that they are not really nanotechnology-related patents—given the requirements and the definition of the nanotechnology field. However, this issue is improving as the B81 (micro-structural technology) classification is now being used more carefully and there is assigned teams that work on this issue. The three clusters are presented in Fig. 3. If a list of ‘nano’ terms is used to collect the required patents, there is a big possibility that unrelated patents will be collected. Moreover, if one attempts to exclude unnecessary patents by utilising such terms as ‘-nanosecond*’, there is a possibility that required patents also will be excluded, as there is a significant number of patent documents which mention nanotechnology-related terms and nanoseconds. It can be argued that there is a possibility of having non-nanotech-related patents or missing nanotech-related patents in the collected data due to the issues stated above. However, this patent search query is an effective method in terms of higher reliability of patent data gathering when compared to other methods. It is accepted that there would be some amount of noise in the collected patent data as there is a significant chance that there are still some patent documents that are not related to the nanotechnology field. Also, there is some chance that a few nanotechnology-related patents may be eliminated if there are any nanotechnology-related patents that are not classified under its own classifications (this is now a lower chance after the introduction of nanotechnology-related classifications in 2004) or there is a possibility that there may be some nanotechnology-related patents that do not consist of any of those selected terms (nano*, quantum*, fullerene*, etc.). To increase the accuracy of this data, additional nanotechnology-related terms should be identified according to the missing terms in the search query. However, the search query results were leading to a saturation period after introducing new nanotechnology-related terms (a high decremental increase in the total number of patent documents after each new term). Moreover, even if the data were optimized further, the results would not be noticeably different given the type of analysis being followed.

**Fig. 4:**
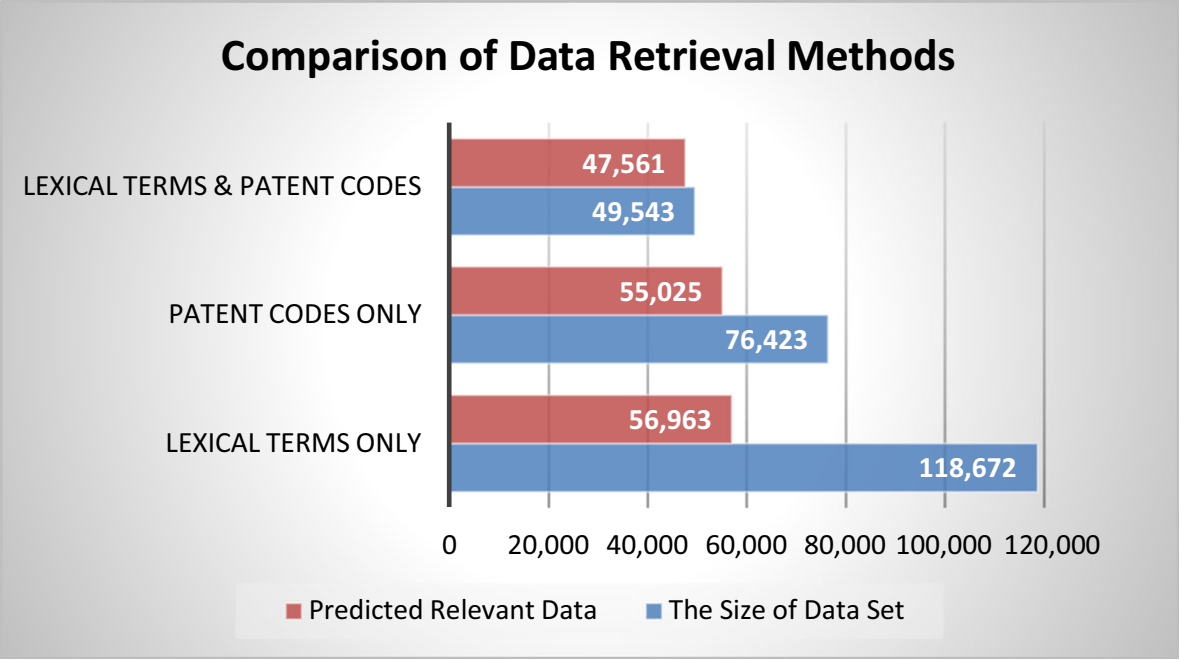
Comparison of relevant data versus size of data set

As shown in Fig. 4, the combination of lexical terms with patent codes result in more relevant data when these three different types of data collection methods are compared. The accuracy of this data collection method is tested by two methods. The first, a sample data (1% of the entire data set) is collected from the actual data set with a purposive sampling for in-depth examination for each three types of data retrieval method. Purposive sampling is completed by putting the search results in more relevant to less relevant results by using Thomson innovation`s “display and sort options” and collecting last 1% (the less irrelevant data). For the first data set with “only lexical term”, the accuracy of the sample was considerably low with almost 48% accuracy that the displayed patent documents that were actually nanotechnology relevant patent documents. The common problem with these patent document results is that there were many statements with nano-related terms in their “claims” section that as the main invention being something that adaptable or usable with nanotechnology materials or technology. The same test is completed with “only patent codes” sample and the results were much better than “only lexical terms” with 72% accuracy. The common problem with the rest of 28% patent documents is that there were many B82 patent coded patent documents there were many inventions about micro level studies or patent documents where the main purpose of it is not about nanotechnology practice or implementation. Finally, the sample data of “lexical terms and patent codes” is tested and the results showed drastically improved results with 96% of accuracy where there were very few irrelevant patent documents. As shown in Table 2, the relevant and the irrelevant data is predicted for all cases. Accordingly, using both lexical and patent code approach leads to a little lose in the size of the data but the retrieved data is much more valid than other two methods.

**Table 2:** Comparison between different types of patent retrieval methods

	The size of data set	The sample data (1%)	Size of relevant data for 1% sample (%)	Size of irrelevant data for 1% sample (%)	Predicted relevant data based on sample results	Predicted irrelevant data based on sample results
Lexical terms only	118,672	1187	48	68	56,963	61,709
Patents code only	76,423	764	72	28	55,025	21,398
Combination of lexical terms and patent codes	49,543	495	96	4	47,561	1982

### Methods for patent data optimisation and analysis

After collecting the patent dataset, there are still certain steps that need to be taken to optimize the data. There are still some unnecessary documents that could not be eliminated in the data collection step and also there are duplicates since patents are granted in different patent authorities by the same companies. However, as mentioned in the previous section, duplicates are very rare in the data since DWPI is used in the patent data collection phase. For those remaining irrelevant documents, TDA software is used to eliminate them and to analyse the optimized document to achieve the required results.

Optimisation is not only followed to eliminate unnecessary data, but it is also used to categorize different fields together, considering the country; the organisation or the technology bases of the patent documents. Moreover, this is the phase where new categories are created to increase the depth of analysis. For example, patents are all grouped into their particular technologies by using patent codes and organisations are categorized into their specific actor category, such as academia and industry. These categories are important for the analysis of this research as academic and industrial collaborations need to be identified and these categories are not directly available on patent documents. For this reason, certain methods are followed to advance the categorisation of patent documents. To this end, certain words are searched, such as university, institution or LLC (limited liability company), in the names of organisations to expedite the categorization process.

Some of these processes are automated by available filters and thesauri that are available in TDA. TDA is used with structured and semi-structured data and it has certain prerequisites for the import of data into the software, such as the format of the document (.txt, .cvs, .xls, etc.) and it has to have the correct filter that corresponds to the database from which the data was compiled. These filters and thesauri are very helpful since there are almost 50,000 patent documents to be categorized and so the data has different fields to be analysed. However, many steps are not fully automated and some patent documents need to be manually grouped together. For that reason, many filters and thesauri are generated specifically for the nanotechnology field. These are specific to those organisations and individuals that operate within the nanotechnology field and they are also specific to special circumstances. For example, there are various cases where some companies work under the umbrella of one large entity, and so these patents are grouped as one organisation as these two organisations influence results as they collaborate closely with one another but, in fact, both entities enjoy the right to use the same patents. To give another example, patent documents show organisations as headquartered in the locale in which patents are granted but, as multinationals, their true locale is elsewhere (e.g. Samsung is a South Korean headquartered, multinational company, but high numbers of their patents are granted in the US). Therefore, manual tagging is followed to categorize organisations into their countries of origin. Accordingly, many new thesauri, scripts and filters are produced for TDA to repeat these studies.

After optimization is completed, various analyses of the data are followed to obtain the required results. In general, these methods are referred to as ‘data mining’, ‘text mining’ or ‘tech-mining’ as explained in a previous section. Some of these methods are of the types, trend, landscape, network, patent portfolio, citation and topological analysis. The common aim of all of these methods is to analyse patent data in an efficient and effective way. As collected nanotechnology patent data is a very large dataset, visualisation and advanced patent data analysis are required. Having visual results facilitates the understanding and interpretation of the data as qualitative analysis on a large patent data set is not possible. After these initial analyses of the visual results, it is then possible to examine the details of the patent data.

In landscape analysis, the frequency of terms in certain sections of the data—such as patent classification codes; patent co-ownership; shared terms in titles and abstracts and citations—were used for clustering the data. This type of analysis is also termed as ‘mapping’ the data, or cluster analysis, as referred to in some other sources. For this, TDA software is used. TDA performs multidimensional statistical analysis to identify clusters and relationships among them. Each cluster is represented by nodes and the size of a node represents the number of patent/publication documents that belongs to it, while its centrality represents how often that particular node occurs with other nodes. The closeness of nodes and their thickness are calculated on the basis of the significance and interrelationship level between each node, which in turn is calculated on the basis of how many of those documents belong to the node and how many of those documents are shared. If there is a high significance between the nodes then the thickness of the line between the two nodes is increased or, conversely, the line is rendered more thinly. To these calculations, co-occurrence matrixes, factor maps and mapping (cross-correlation or auto-correlation) is added. By following these types of analysis, successfully collaborating organisations are identified. Clusters and the network structure of the nanotechnology field are illustrated. Many key issues—such as network structure and type; central/dominant actors; national differences and many other aspects related to this research—are identified within this part of the analysis. However, TDA can only identify relationships between organisations and cannot describe the exact type of relationship between them. For that reason, a qualitative analysis outside of the software should be followed to grasp the details of the collaboration. This can be considered as a weakness and is explained further in the section where the limitations of the patent analysis are addressed.

As a result, 49,544 individual nanotechnology patents were obtained for the period from 1970 to 2012. The obtained results were imported into the TDA and to validate the results further. The duplicate results were eliminated and variations of company, inventor, institutes and university names were unified where they appeared as separate patent assignees. After the dataset was cleaned and prepared, various functions were utilized using the same TDA tool to generate the required analysis.

## Analyses of patent information: nanotechnology patent collaborations

### Patent collaboration between actors

In general, the progress of nanotechnology patenting activity appears to be very promising for commercial activities. There are 73,096 inventors; 29,884 organisations and 68 countries involved in nanotechnology patenting activity. There are 49,544 patented inventions, of which 29,217 are owned by corporations; 10,787 by academic organisations (universities and other institutions); 14,164 by inventors and 1887 by governments. The total number is higher than the actual patent number because there are a number of shared patents among different organisations (see Table 3). There are 1784 patents that are shared by corporate and academic organisations. Table 3 also indicates the significance level of linkages according to the ratio of collaborative patents to the total number of patents for each type of collaborating actor. The results indicate that over 20% of patents are generated as a result of collaborated patents based on co-ownership analyses where a single patent is owned by more than one types of actor when compared to those patents that are owned by a single actor type. However, the results may be misleading for the following reasons: first of all, looking at the significance level of linkages, the highest significance of co-owned patents appears to be between corporations and inventors, which is higher than the significance level of the academia-inventors linkage. However, this may indicate internal R&D in the nanotechnology field. Likewise, the linkage between inventors and academia may indicate patents generated within a university’s research facilities. Although, the significance level of academic-industrial collaboration is not very high, it is significant for the technology transfer process as almost 20% of patents are the result of academic-industrial linkages. Secondly, these results may be influenced by the type of agreements whereby academic-industrial collaborative inventions are only licensed to industrial players.

**Table 3:** Patent collaborations between actors

	Government (1887)	Academic (10,787)	Inventors (14,164)
Corporate (29,217)	163 (0.005)	1784 (0.045)	6850 (0.158)
Inventors (14,164)	406 (0.025)	2050 (0.082)	
Academic (10,787)	194 (0.015)		

Table 3 also shows government linkages with other actors. The results show a very low significance for this type of patent co-ownership, which may illustrate the effectiveness of the Bayh–Dole Act and similar legislation as governments transfer ownership of patents resulting from federally-funded research to colleges, universities, or other institutions which have been contracted to carry out the research. Moreover, after reviewing governmental-related patent documents, it was found that these linkages are not only due to funding systems but that there are actual research collaborations where military or other types of government-based organisations are involved. For that reason, new patent ownership legislation appears to be working, and this is very important as it is better to transfer ownership of inventions back to the inventors and organisations who fundamentally understand the commercial potential of their patents.

Having identified noteworthy relationships between academia and corporations in the nanotechnology field, top collaborative academic and industrial actors are examined in Table 4. As shown below, the most significant linkage is between Tsinghua University (Qinghua University as it appears on tables and figures and will be referred as Tsinghua University form now on) and Foxconn (Hon Hai Precision as it appears on tables and figures and will be referred as Foxconn form now on). The Japanese nanotechnology innovation system also appears to function well—given the fact that there are many linkages between industrial and academic organisations. In South Korea, there is a better level of collaboration exists between governmental organisations and Samsung compared to some of the top nations. In the case of the US, it seems that there are few patents shared between top organisations; however, there appears to be some collaboration between MIT and top organisations, such as HP and IBM (MIT shares a single patent with each of these organisations).

**Table 4:** Academic and corporate collaboration matrix

#Records	Cooperate	#Records									
		600	594	551	496	408	358	318	275	263	249
		Academic									
		Dokuritsu Gyosei Hojin Sangyo Gijutsu SO	Univ California	Japan Sci and Technology Agency	Univ Qinghua	Dokuritsu Gyosei Hojin BusshitSu Zairyo	Korea Adv Inst Sci and Technology	Univ Seoul Nat Ind Found	Massachusetts Inst Technology	Commissariat Energie Atomique	CNRS Cent Nat Rech sci
1258	Samsung Electronics co Ltd		6				7	30			
689	NEC Corp	11		48							1
686	Fujitsu Ltd	2									
657	Hon Hai Precision Ind Co Ltd				417						
608	Int Business Machines Corp								1		
571	Matsushita Denki Sangyo KK	2		1							
500	Sony Corp										
483	Toshiba KK	2		12							
477	Hitachi Ltd	1		5							
439	Canon KK										
438	Fuji Photo Film Co Ltd										
435	Nippon Telegraph and Telephone Corp			1							
414	Hewlett-packard Dev Co LP		1						1		
386	Sharp KK	1									
298	Mitsubishi Electric Corp	5									

**Fig. 5:**
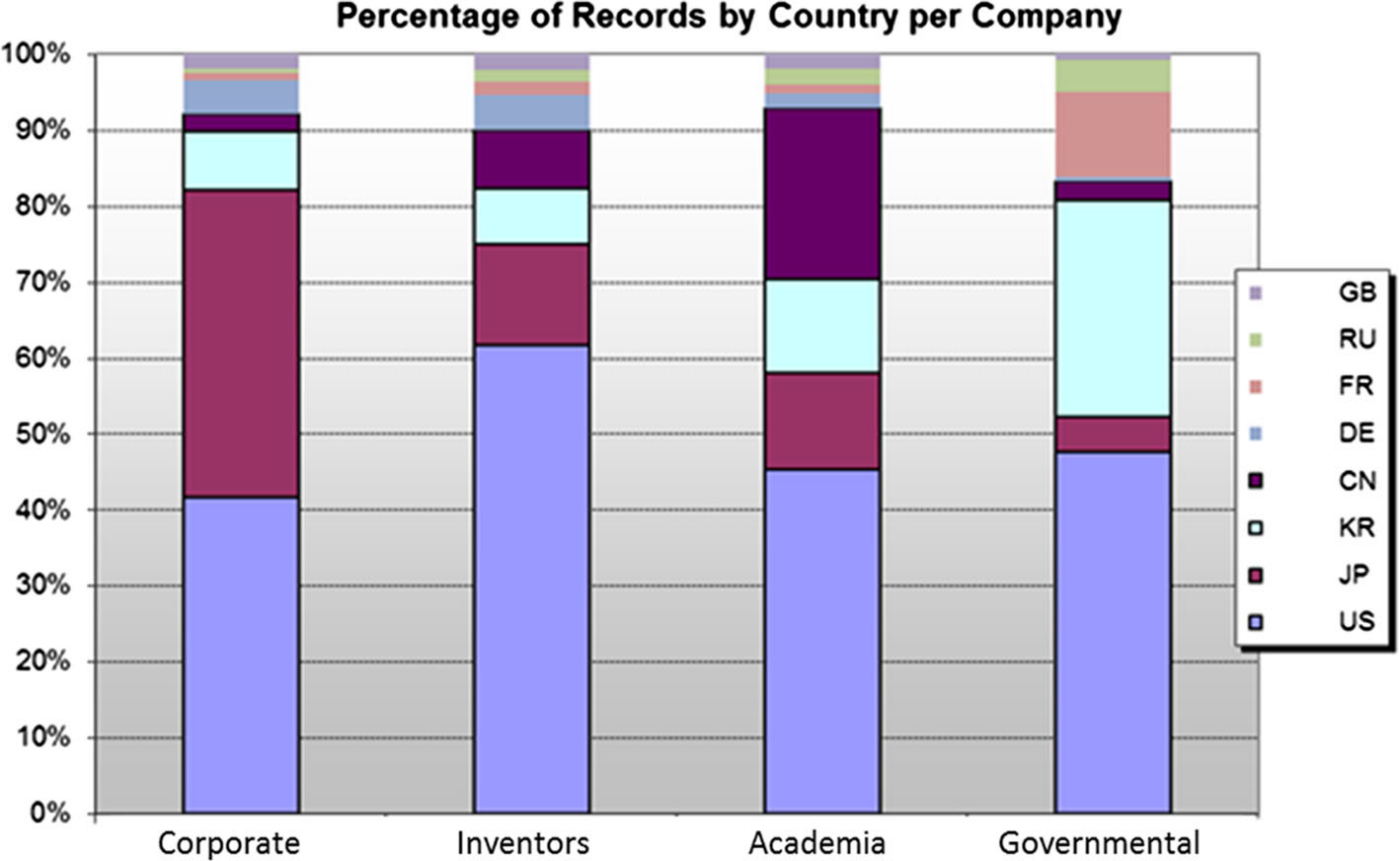
Countries involvement based on type of actors

At the organisational level, Japan appears to have the highest degree of organisational involvement—given the linkages between academia and corporation. However, this may not reflect Japan’s true level of involvement in the nanotechnology field. For that reason, global nanotechnology patents have been categorized into four different areas, namely: academic, industrial, inventors and governmental. As shown in Fig. 5, even though Japan has a smaller number of nanotechnology-related patents, it still has almost the same number of patents as the US at corporate level. Individual inventors in the US appear to have the highest degree of patent ownership, followed by Japan. It can be concluded that China’s rapid progress is largely due to academic players. In the case of Korea and France, there is a more successful level of collaboration between governmental organisations and firms compared to some of the leading nations.

Since the technology diffusion period of many technologies is becoming progressively shorter due to strong networks; systematic approaches and developed information and communication technologies, the increased number of nano-patents may lead to the commercialisation stage in the near future. Additionally, the availability of almost 50,000 granted nano-patents within the maximum patent grant period of 20 years suggests the highly commercialised era of nanotechnology is imminent. However, as mentioned by various analysts (Kronz and Grevink 1980; Suzuki 2011), only a few patents have commercial viability, so only some of these patents will be turned into innovative products.

### Analyses of the key patent strengths

This section examines where countries’ competitive strength derives from within the nanotechnology field and who the country-specific dominant players are. In this section, for each country, some categories are chosen to differentiate their involvement, such as the duration of years they have been involved in nanotechnology field; their top organisations and their involvement in the last 3 years (to show how active they are in nanotechnology patenting and research, based on their recent involvement). Looking at Table 5, it can be seen that almost every country appears to have at least one strong technology domain that is not a highly competitive point for another country (or one particular technology may not be the key capabilities of a country as they may have a lower ratio of patents compared to their total number of nanotechnology patents). This is found to be mainly related to the leading organisations’ involvement in their regions (for example, NEC’s involvement in laser-related technologies). However, this was a different case in regions where SMEs have a high role, such as in Germany.

**Table 5:** Nanotechnology patent strengths

Country	US	JP	KR	CN	DE	FR	RU	GB	TW
Number of records	21,648	15,257	4830	4018	2021	1066	893	850	748
Top organizations	Samsung Electronics Co Ltd [870]; Univ California [589]; Int Business Machines Corp [550]	NEC Corp [681]; Fujitsu Ltd [650]; Dokuritsu Gyosei Hojin Sangyo Gijutsu So [594]	Samsung Electronics Co Ltd [1215]; Korea Adv Inst Sci and Technology [351]; Univ Seoul nat Ind Found [267]	Yang M [908]; Foxconn [500]; Univ Tsinghua [489]	Siemens AG [133]; Infineon Technologies AG [109]; Fraunhofer Ges Foerderung Angewandten Ev [90]	Commissariat Energie Atomique [249]; CNRS Cent Nat Rech Sci [204]; L’oreal SA [122]	Sokolov S V [20]; Kamenskii V [19]; Moscow Lomonosov Univ Chem Faculty [15]	Toshiba [46]; Isis Innovation Ltd [34]; Imperial Innovations Ltd [32]	Ind tech res Inst [201]; Foxconn [141]; Univ Taiwan Nat [42]
Year range	1967–2011	1970–2011	1991–2011	1997–2011	1968–2010	1963–2010	1993–2011	1969–2010	1997–2010
Percentage of records in last 3 years	12% of 21,648	9% of 15,257	24% of 4830	40% of 4018	7% of 2021	9% of 1066	38% of 893	8% of 850	22% of 748
Top technology terms	A12-W14: Polymer applications [1959]; D05-H09: fermentation industry—testing and detection [exc. bacteria, fungi, viruses] [1679]; B04-C03: natural or genetically engineered products polymers [1248]	V08-A04A: semiconductor laser [2001]; E05-U03A: carbon nanotubes → single walled [1308]; U12-A01B1B: Semiconductor details of laser body → Quantum well semiconductor laser [1060]	A12-W14: Polymer applications [812]; U11-A14: semiconductor materials and processing → material—Nano-structural materials [353]; U12-B03F2A: devices and thick/thin film and organic semiconductor devices—nanostructural devices [327]	B04-A10: natural products (or genetically engineered), polymers → plant extracts [851]; B04-A08: natural products (or genetically engineered), polymers → plant divisions and whole plants[789] B04-A09: plant parts derived from specific plant species [731]	D05-H09: fermentation industry—testing and detection [exc. bacteria, fungi, viruses] [102]; J04-E04: chemical physical processes apparatus → catalysts [95]; A12-W14: polymer applications [94]	A12-V01: polymer applications → medicines, pharmaceuticals [69]; E05-U03: carbon nanotubes [66]; A12-V04C: polymer applications → cosmetics, toilet requisites → skin requisites [64]	U12-B03F2: devices and thick/thin film and organic semiconductor devices—nanostructures [77]; A12-W14: polymer applications [54]; E31-U01: nonmetallic elements, metalloids and compounds → nanoparticles [44]	V08-A04A: semiconductor laser [70]; D05-H09: fermentation industry— testing and detection [exc. bacteria, fungi, viruses] [65]; U12-E01B2: semiconductor body with quantum wire, wells, superlattice [65]	E05-U03: carbon nanotubs [98]; A12-W14: polymer applications [91]; U12-B03F2: devices and thick/thin film and organic semiconductor devices—nanostructures [76]

Even though some countries such as China (1997) and Korea (1991) are latecomers to nanotechnology patenting and research, they managed to emerge as two of the leading nations. If the number of technology terms is considered per country, the US appears to have the highest involvement in nanotechnology polymer technology (1959 patents) and the US is the only country that contains nanotechnology patents that are related to natural/genetically engineered product polymers within the top three nanotechnology terms. Japan’s leading nanotechnology-related field is semiconductor laser technology and NEC appears to be the dominant player in Japan with 681 patent documents. Another key nanotechnology patenting area for Japan is carbon nanotube technology. Carbon nanotubes are nanostructures that have a great use in various fields; in different forms and Japan can gain great benefits from this area once it is commercialised and if they can maintain their leading position in their patenting activities. In the last 3 years, China has emerged as being in a significant position as it has presented the greatest growth with 40% and the country is the newest nanotechnology player when compared to other leading countries. For China, the dominant organisation appears to be Hon Hai Precision (Foxconn), the Taiwanese-based global manufacturer and the largest exporter in China.

**Fig. 6:**
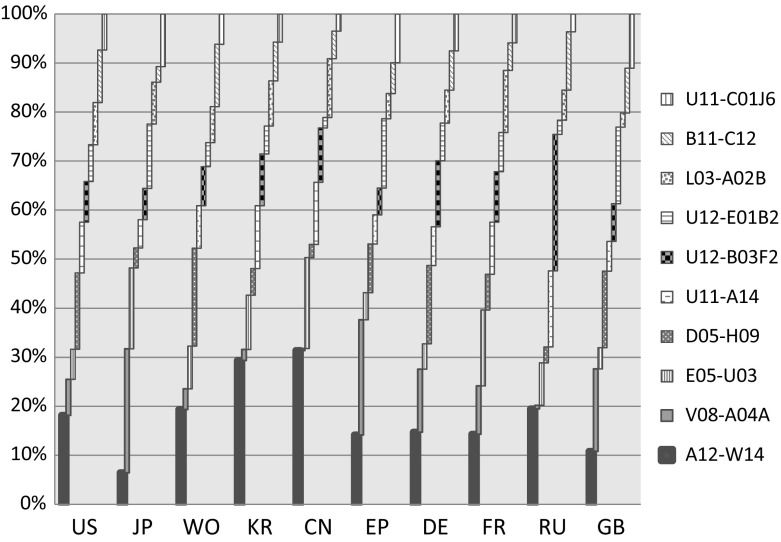
Strength of nanotechnology domains by country

Having mentioned the key technologies for each country, the top sub-categories in the nanotechnology field are analysed to reveal the number of patents which have been granted for each technology field by which country. In general, the top technology terms for each country can be identified, but Fig. 6 allows the analysis of the top ten technology patenting fields with regard to the number of patent documents that have been granted for leading countries in nanotechnology. The novelty of this analysis is that it presents all the dominant countries in a specific technology field and, at the same time, the weaknesses of countries in their patenting activity in a specific technology. For example, the US, the UK and Germany are very dominant in carbon nanotube compositions and structures (E05-U03) and Japan is highly dominant in nanotechnology-related, semiconductor laser research and patents (see “Appendix” for information on Derwent manual code). These patents are essential for many kinds of research because these are the patent documents that contain the core notions of research in nanotechnology. These patents can be an obstacle for some countries in getting involved in some of nanotechnology fields.

The considerable differences between countries are due to the different interests of public and private organisations or due to the availability of sufficient funds for that specific field. For example, the reason why Japan is far ahead in semiconductor laser technology compared to other countries is because of the high interest and investment of Japanese companies in this field. The companies involved include ones such as NEC, Fujitsu, Hitachi, Toshiba, Mitsubishi Electric and NTT. This technology is applied in many different fields including network devices, printers and sensors. Considering the huge interest of NEC in terms of their current technology focus, they are motivated to invest in semiconductor nanotechnology lasers; thus, Japan appears as the leading country in this field. Also, Japanese academic organisations, such as Dokuritsu Gyosei Hojin Sangyo Gijutsu (National Institute of Advanced Industrial Science and Technology), receive a lot of support from government and private organisations to enable them to focus on this type of research.

### Countries and organisations involvement in patent collaboration

**Fig. 7:**
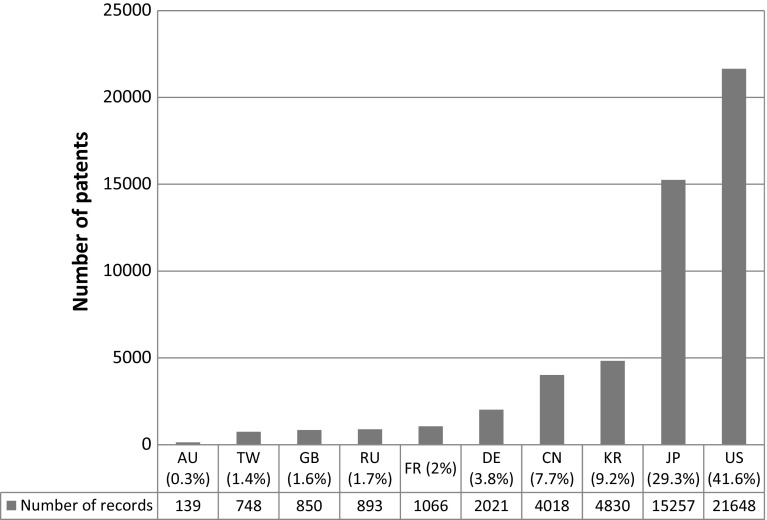
Nanotechnology patents per country

As shown in Fig. 7, the US, with 41.6% of the overall patents, is still the leading country in the nanotechnology field. However, it appears that Asian players (Japan 29.3%; Korea 9.2% and China 7.7%) are catching up and the Asian region has the highest number of patents in total. The increasing importance of Korea and China as players in the nanotechnology field can be considered as a threat for the US and Japan. In the EU region, Germany, France and the UK play key roles in nanotechnology patenting activity but they are far behind the Asian players and the US. With regard to the above analysis of the number of patent records per country, it seems that nano-patenting activity did not spread to other EU countries and the growth rate of the EU number of patents is very slow. Russia and Taiwan emerge as being important regions for nanotechnology. It can be said that there was a significant increase in the number of Chinese patents in 2001, though one reason why China did well as a country was because the inventor Yang Mengjun was granted 908 patents that year (see Fig. 6). His nanotechnology research focuses on nano-foods, specifically on ancient Chinese medicinal herbs which he reduces to the nanoscale to increase the efficiency of the formulation. All of his patents have been granted by the Chinese Patent and Trademark Office. Yang Mengjun’s inventions were found to have limited applicability to other nanotechnology-related activities. Earlier in the analysis, in the “Analyses of the key patent strengths” section China seems to be the leading developer of nanotechnology applications in the area of natural products, which is due to the huge amount of patents granted by the same Chinese scientist.

**Fig. 8:**
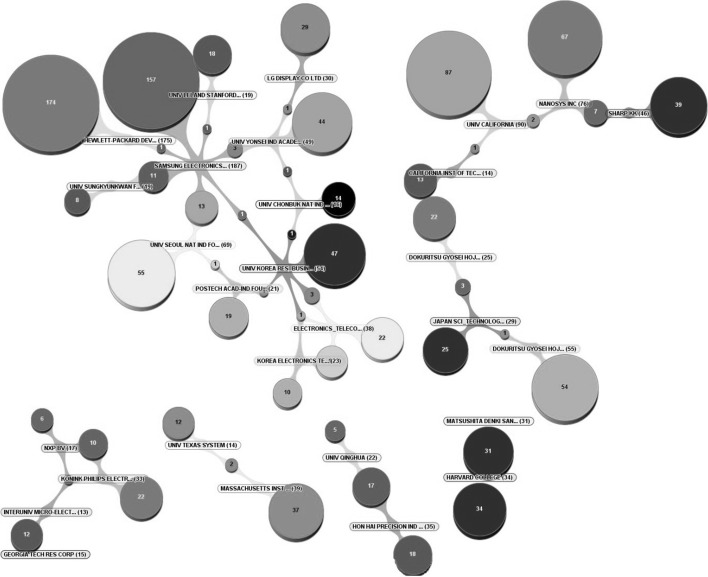
Nanotechnology patent collaboration through countries and organisations

Having examined countries’ progress in the nanotechnology field, the patent collaboration between organisations in the nanotechnology field with regard to shared and collaborative patents is analysed—as shown in Fig. 8. It is possible to capture the collaboration level of organisations; the linkages of organisations within/outside their establishment in whichever country they operate and their collaboration with other actors within the nanotechnology innovation system. After analysing the networks of the top 250 nanotechnology organisations (see Fig. 8), the strongest linkage was found between Foxconn and Tsinghua University as was also mentioned earlier in Table 5. These two organisations follow research on basic and applied nanotechnology, to create new-technologies and provide impetus to the commercialisation of nanotechnology activities. They have mainly focused on the applications of nanotechnology in the electronics’ industry and one of their areas of expertise is carbon nanotubes. There is no other organisation that shares any patents with either Foxconn or Tsinghua University. This shows that strong linkage between two large organisations creates a barrier for other organisations to become involved in such collaboration.

The second strongest bond between two organisations—considering the ratio between number of patents held by the individual organisations and the patents that have been granted collaboratively—is between Samsung and Sungkyunkwan University of Korea, as they share 37 patents within the nanotechnology field. This type of academic-industrial relationship mostly appears to be in Asian region where industrial actors establish their physical locations and institute exclusive agreements where IP rights are shared. This appears to be a better model for long-term innovation development, rather than just one time technology transfer and acquirement of the new knowledge. Looking this at a macro level, this can be related to the governments’ involvement in these regions to support and encourage by technology funds and policies to enhance these types of relationships. Also, only the largest firms have capacity to invest at this level but many key actors in this field still do not appear to believe in the advantages of long-term and substantial investments together with academic actors. The relationship between Samsung and Sungkyunkwan University is similar to that of Foxconn and Tsinghua University, but the Korean players are more open to other organisations, as they also collaborate with other organisations in this field.

As shown in the Fig. 8, US, Korean and Japanese actors present significant linkages within their own regions. If these regional nanotechnology innovation systems are compared, it can be said that the Japanese innovation system appears to have the strongest and highest number of linkages between organisations. Another powerful nanotechnology innovation system is in the Korean region. Even though the Korean players became involved in nanotechnology long after other national players, the Korean innovation system has a robust cluster and emerges as one of the most prosperous. Most of their linkages are within their own national innovation system, which appears to be highly centralized around the key Korean player, Samsung.

A different characteristic can be found within a different research cluster that embraces US and French-based organisations. This is the only nanotechnology research cluster that has strong relationships with regard to patenting activity at the international level. Some of the US and French research institutes and universities collaborate with each other, such as the collaboration between the California Institute of Technology (USA) and the Centre National de la Recherche Scientifique, CNRS (France). The linkage between Motorola and Freescale Semiconductors is strong in the US. Even though this cluster is the significant cluster with the characteristics of a strong international linkage, it seems that key patenting activity still remains within their respective national boundaries.

## Discussions and implications

This paper contributes to information systems’ management by offering an integrated taxonomy development and its successful implementation in analysing nanotechnology patent information. This study has retrieved 49,544 patents within the nanotechnology field using the Thomson innovation database, with the subsequent analyses utilising TDA software, which makes the results increasingly valid and reliable, as this data mining software allowed the data to be cleaned further to eliminate unnecessary patent documents, such as duplicates within systems of innovation; identifying emerging actors and their patent collaborations constitutes a worthy contribution to patent information management. With regard to adopting the TEN approach, networks within the nanotechnology system showed that boundaries of organisations’ interactions are not limited to a national level, so it is not possible to limit this field of study to certain sectors either. However, a great weakness of the nanotechnology system is that the linkage between the S–T poles and the market pole is not strong enough in terms of patenting activities. The research has showed some strong linkages, such as between Foxconn and Tsinghua University, but this type of collaboration is rare. The weakness in collaboration between the S–T poles and the market pole may be one reason why the nanotechnology field is not in its highly commercialized stage. Strengthening the linkages between scientific and corporate actors may eliminate many barriers and accelerate the diffusion of technology in the commercial and scientific fields. The focus of nano-science and technology development (S–T poles) corresponds to the importance of these areas in the commercial domain (market pole).

This paper makes a methodological contribution to the field by improving patent search queries and by offering a comparison of patent databases to enable active and potential specialists to select the most convenient option for their patent-related studies. Moreover, this study critiques the patent collection methods of previous studies and defends the reliability and validity of the method used here with the illustrated figures. Furthermore, this study illustrates how data mining tools, such as VantagePoint or TDA, can be used to analyse patent documents to retrieve information. It goes further by illustrating the possible interpretation of these descriptive findings, allowing further analysis. It should be stressed that decisions on data mining tools and patent data bases were made purely on the basis of the requirements of this study. Although a combination of patent classification codes with lexical queries appears to be the most fruitful technique, lexical queries may be more appropriate in cases such as Porter et al. (2008) and Huang et al. (2003). For that reason, it should be stated that the chosen patent database; the search query method or the data mining tool may not be the best option for another study due to various reasons such as the amount of patent authority coverage or field of study where a different data retrieval approach is more suitable.

The analysis of nanotechnology patenting activity presented the recent technology development and diffusion trends. International profiles provided useful details, such as changing trends appear in the nanotechnology field. Existing studies showed that the US and Japan were leading all other countries, but this new patent data analysis shows that Korea, China, Russia and Taiwan are the possible top-ranking countries for nanotechnology. Moreover, this research has presented country-based, key technology domains and dominant players within those countries. Asian companies’ involvement has emerged as being very successful as Samsung has become the leading organisation; including NEC and Fujitsu—2nd and 3rd respectively—and Hon Hai Precision holds 4th place in the nanotechnology field. The findings scrutinized the top actors’ profiles and their linkages, where Asian players reveal their noticeable roles.

This research offers an innovative insight into various organisational relationships in terms of patent collaboration. By using TDA software, it has presented different collaborations at national and international level. Nations such as Korea and Japan are found to be highly collaborative. The US is in collaboration with France. China presented a great illustration of an effective collaboration in patenting activity and co-inventorship between academic and corporative organisations. It was found that the strongest linkage is between Foxconn and Tsinghua University with 417 granted patents, which were supported by their establishment of the Tsinghua–Foxconn Nanotechnology Research Centre. The findings expose that several Korean, Japanese and Chinese companies belong to the largest commercial players in the technology pole of the global socio-economic network. Therefore, it would be useful to adopt strategies that could facilitate in building a network platform for sharing or exchanging nano-expertise; key technologies and nano-information across the region.

The implication of this study is that the new integrated taxonomy provides a reliable and efficient tool for accessing accurate nanotechnology patent information that enables active and potential participants in this field to gain from: (1) Knowledge of changing trends in nanotechnology at country and organisational level, for example the changing role of Asian countries and the increasing importance of Korean organisations; (2) Evaluation of the competition and core competences of nanotechnology organisations and countries; (3) Examination of existing and future technologies within the nanotechnology field to examine their potential commerciality; linkages of patent classes with various industries and the interconnection of various nanotechnology patents amongst different technology areas; (4) Analyses of national and international patent sources and collaboration of various organisations present strong linkages and the dominant players within this field.

The limitations and gaps of existing studies in searching accurate patent information led to the initial idea of a more comprehensive analysis on nanotechnology actors and their linkages that have so far been accomplished in this paper. There are many other relationships that can be analysed with nanotechnology patent analysis. Future studies could explore different technology domains and their relationships with each other at country and international levels. This quantitative study could be taken to a qualitative level by analysing the technology’s claims and abstracts of each patent to see how a specific sub-nanotechnology category is linked with corporations. The quality of patents rather than the quantity of patents could be analysed in various ways; for instance, by looking at citation linkages between patents. A statistical analysis of the relationships pertaining between commercialised patents and the latest granted patents could be performed to examine various relationships and forecasts between inventions and innovations. It is expected that further knowledge about understanding patent information systems can be obtained through further research in order to increase its robustness.

## Appendix: Information on 10 Derwent manual code with highest number of patent records

**Table:**

Manual code	Detailed information
A12-W14	Plasdoc/polymer applıcatıons–other applıcatıons/nanotechnology
V08-A04A	Semiconductor laser
E05-U03	Single walled carbon nanotube
D05-H09	Testing and detection of microbiology, laboratory procedures
U11-A14	Semiconductors and electronic circuitry/semıconductor materıals and processıng/materıals/nano-structural materıals
U12-B03F2	Semiconductor structure in nanoelectromechanıcal device/system
U12-E01B2	Semiconductor structure, manufacture in nanoelectronic device/system
L03-A02B	Carbon and graphite conductors
B11-C12	Farmdoc/processes, apparatus/general process, pharma applications
U11-C01J6	Heterojunction, superlattice structures, quantum wells, wires, boxes manufacture
